# Overcoming Immunotherapy Resistance by Targeting the Tumor-Intrinsic NLRP3-HSP70 Signaling Axis

**DOI:** 10.3390/cancers13194753

**Published:** 2021-09-23

**Authors:** Balamayooran Theivanthiran, Tarek Haykal, Linda Cao, Alisha Holtzhausen, Michael Plebanek, Nicholas C. DeVito, Brent A. Hanks

**Affiliations:** 1 Department of Medicine, Division of Medical Oncology, Duke University, Durham, NC 27708, USA; bala.theivanthiran@duke.edu (B.T.); tarek.haykal@duke.edu (T.H.); linda.g.cao@duke.edu (L.C.); michael.plebanek@duke.edu (M.P.); nicholas.devito@duke.edu (N.C.D.); 2 Lineburger Comprehensive Cancer Center, University of North Carolina at Chapel Hill, Chapel Hill, NC 27599, USA; alisha_holtzhausen@unc.edu; 3 Department of Pharmacology and Cancer Biology, Duke University, Durham, NC 27708, USA

**Keywords:** NLRP3 inflammasome, HSP70, adaptive immunotherapy resistance, granulocytic myeloid-derived suppressor cells

## Abstract

**Simple Summary:**

The tumor-intrinsic NLRP3 inflammasome is a newly recognized player in the regulation of tumor-directed immune responses and promises to provide fresh insight into how tumors respond to immunotherapy. This brief review discusses recent data describing how activation of the tumor-intrinsic NLRP3 inflammasome contributes to immune evasion and what this pathway may provide to the field of immuno-oncology both in terms of pharmacologic targets capable of boosting responses to checkpoint inhibitor therapies and predictive biomarkers indicating which tumors may be most susceptible to these new therapeutic strategies.

**Abstract:**

The tumor-intrinsic NOD-like receptor family, pyrin-domain-containing-3 (NLRP3) inflammasome, plays an important role in regulating immunosuppressive myeloid cell populations in the tumor microenvironment (TME). While prior studies have described the activation of this inflammasome in driving pro-tumorigenic mechanisms, emerging data is now revealing the tumor NLRP3 inflammasome and the downstream release of heat shock protein-70 (HSP70) to regulate anti-tumor immunity and contribute to the development of adaptive resistance to anti-PD-1 immunotherapy. Genetic alterations that influence the activity of the NLRP3 signaling axis are likely to impact T cell-mediated tumor cell killing and may indicate which tumors rely on this pathway for immune escape. These studies suggest that the NLRP3 inflammasome and its secreted product, HSP70, represent promising pharmacologic targets for manipulating innate immune cell populations in the TME while enhancing responses to anti-PD-1 immunotherapy. Additional studies are needed to better understand tumor-specific regulatory mechanisms of NLRP3 to enable the development of tumor-selective pharmacologic strategies capable of augmenting responses to checkpoint inhibitor immunotherapy while minimizing unwanted off-target effects. The execution of upcoming clinical trials investigating this strategy to overcome anti-PD-1 resistance promises to provide novel insight into the role of this pathway in immuno-oncology.

## 1. Introduction

Much of our understanding of anti-tumor immunity is based on studies focused on the adaptive immune response to cancer. However, our insight into the interactions between the tumor and the innate immune system and its role in controlling tumor metastasis has remained incomplete. There has been a significant amount of work in recent years describing and characterizing a key role for the inflammasome in regulating innate immunity, predominantly in the context of inflammatory and autoimmune diseases [1,2]. The canonical inflammasome describes a macromolecular complex comprised of a scaffolding protein that, upon activation and oligomerization, induces the activation of caspase-1. Caspase-1, in turn, cleaves and converts the inflammatory cytokines, pro-IL-1β and pro-IL-18, into their active forms and facilitates their secretion. This same process drives the cleavage and activation of the pore-forming protein, gasdermin-D, which enables the secretion of these cytokines as well as danger-associated molecular patterns (DAMPs) in the presence or absence of a process of inflammatory cell death known as pyroptosis [2,3]. NOD-like receptor family, pyrin-domain-containing-3 (NLRP3), also known as NALP3 or cryopyrin, represents the most well-studied inflammasome, and its role in inducing inflammatory responses is broadly recognized [4,5,6]. While the NLRP3 inflammasome has been demonstrated to respond to a wide variety of stimuli, including various molecules indicative of alterations in cellular homeostasis, it is also regulated by a complex network of pathways that modulate its signaling capacity through an array of post-translational modifications, including phosphorylation and ubiquitination [7]. Importantly, several gain-of-function mutations in the *NLRP3* gene, as well as its regulators, have been identified and associated with various clinical inflammatory syndromes [1,8]. It is this association between NLRP3 and these inflammatory conditions, which has also prompted the development of various pharmacologic inhibitors to suppress the activity of the NLRP3 inflammasome and manage the symptoms of these rare disorders [9,10]. While much of our understanding of NLRP3 biology has been derived from studies in myeloid cell populations, such as macrophages and dendritic cells, its tumor-intrinsic role has historically been overlooked. After briefly discussing prior studies implicating a role for the NLRP3 inflammasome in both cancer progression and the regulation of the adaptive immune system, this review then focuses on developments that indicate an important role for the tumor-intrinsic NLRP3 inflammasome in acquired immunotherapy resistance via the induction of heat shock protein-70 (HSP70) release and the ultimate recruitment of granulocytic myeloid-derived suppressor cells (PMN-MDSCs). Additional discussion is provided regarding the influence that the genetics of this inflammasome pathway may have on immunotherapy resistance and in the identification of those tumors more likely to be responsive to the pharmacologic inhibition of the tumor NLRP3-HSP70 signaling axis.

## 2. NLRP3 in Cancer

The NLRP3 inflammasome has been implicated in promoting the progression of several malignancies by influencing the intrinsic invasiveness of the tumor while also promoting the process of epithelial-mesenchymal transition (EMT) [11,12,13,14,15,16,17,18,19,20]. Indeed, NLRP3 has been shown to be overexpressed by many cancers, while elevated levels of tumor NLRP3 expression have also been associated with an inferior clinical prognosis [15,21,22,23]. This is consistent with data showing *NLRP3* amplification across several solid tumor types, implicating *NLRP3* as a potential oncogene [24]. However, the underlying intrinsic pathways linking NLRP3 with tumorigenesis are still poorly described. Interestingly, one study has identified *NLRP3* exonic mutations associated with FLIP-mediated anti-apoptosis in ~16% of lung adenocarcinomas [25]. NLRP3 activation has also been suggested to contribute to myelodysplastic syndrome by triggering enhanced nuclear translocation of β-catenin and an increased expression of Wnt/β-catenin target genes in hematopoietic stem cells [26]. Still, it is unknown whether NLRP3 activation contributes to tumor de-differentiation via this same impact on the β-catenin signaling pathway in solid tumors. While pyroptosis has been shown to be suppressed in response to NLRP3 activation in certain tumor types, thus promoting tumor cell survival, it is unclear whether the development of this endpoint depends upon the specific stimulus [27]. Mechanisms determining the cell’s fate of pyroptosis versus a state of inflammasome-dependent hypersecretion in tumors following NLRP3 activation would be expected to significantly alter the physiologic impact of this pathway [3].

In addition to its tumor-intrinsic properties, studies have described a role for NLRP3 in regulating the accumulation of immunosuppressive myeloid-derived suppressor cells (MDSCs) within the tumor microenvironment. This is exemplified by cancer-associated fibroblast (CAF) expression of NLRP3, which contributes to tumorigenesis and metastasis in several breast cancer models, including the syngeneic PyMT-derived mammary tumor cell lines, AT3 and Met-1, and the 4T1 mammary carcinoma cell line [28]. This study showed NLRP3 in CAFs to mediate IL-1β-dependent recruitment of both CD11b^+^Ly6C^lo^Ly6G^+^ PMN-MDSCs and CD11b^+^Ly6C^hi^Ly6G^−^ monocytic MDSCs into the tumor bed depending on the genetic background of the tumor model. These findings are in line with previous work implicating a role for host NLRP3 in suppressing the efficacy of a dendritic cell-based cancer vaccine by recruiting CD11b^+^Gr-1^+^ MDSCs into tumor tissues [29]. Further studies have similarly described the periodontal pathogen, *Porphyromonas gingivalis*, as a driver of colon cancer by promoting NLRP3-mediated accumulation of CD11b^+^ myeloid cells into the colonic epithelium [30].

## 3. Tumor-Intrinsic NLRP3 and Its Regulation

While the regulation of the NLRP3 inflammasome has been well examined within various myeloid populations of the innate immune system, very few studies have investigated the tumor-intrinsic properties of NLRP3. It has remained unclear what biological processes NLRP3 may regulate in tumor cells and whether these processes may be different from its function in myeloid cell populations. In addition, it is not understood whether there are tumor-specific pathways that uniquely modulate NLRP3 function relative to the regulatory networks that have been shown to control NLRP3 activity in myeloid cells. However, there are newly available reports that have begun to provide some insight into NLRP3 regulation in cancers. A recent study found Kras^G12D^ oncogene-driven myeloproliferation to be dependent upon NLRP3 inflammasome activation [31]. This work revealed Kras^G12D^ induction of NLRP3 activation to rely upon RAC1-mediated accumulation of reactive oxygen species (ROS) in myeloid leukemia cells. This NLRP3 regulatory mechanism is similar to what has been described for insulin-like growth factor-I shown to induce NLRP3 activation via ROS accumulation in HeLa cervical carcinoma cells [32]. Further studies have also demonstrated a pro-tumorigenic role for NLRP3 under the regulation of interleukin-1 receptor-associated kinase-1-mediated JNK1/2 phosphorylation in hepatocellular carcinoma cell lines as well as the control by estrogen receptor signaling in endometrial carcinoma cell lines and breast carcinoma cell lines [33,34,35]. Additional reports have described roles for microRNA-22 (miR-22), miR-135a, and miR-233 in negatively regulating NLRP3 expression while suppressing the progression of an oral squamous cell carcinoma model, a pancreatic cancer model, and a breast cancer model, respectively [36,37,38]. Future studies to uncover tumor-selective regulators of the NLRP3 inflammasome may allow for the development of pharmacologic agents that avoid impacting myeloid cell inflammasome activity and thus circumvent the development of potential off-target toxicities.

## 4. NLRP3 and Anti-Tumor Immunity

As opposed to its reported role in the innate immune system, NLRP3 inflammasome-mediated regulation of adaptive immune responses to cancer has been less well-described [39]. Prior work has supported a role for NLRP3 in tumor immunosurveillance, including studies showing the NLRP3 inflammasome to upregulate PD-L1 expression and suppress the generation of T cell responses in diffuse large B cell lymphoma while also driving metastatic progression in the B16/F10 melanoma model by inhibiting NK cell activity [40,41]. More recent studies have shown systemic pharmacologic inhibition of NLRP3 to increase effector CD8^+^ T cells and suppress both CD4^+^FoxP3^+^ regulatory T cells and CD11b^+^Ly6G^+^Ly6C^lo^ PMN-MDSCs in a transgenic *Tgfbr1/Pten* 2ccKO model of head and neck squamous cell carcinoma [42]. These data are also consistent with studies performed in a transgenic p48^Cre^;LSL-Kras^G12D^ model and an orthotopic Pdx1^Cre^;LSL-Kras^G12D^;Tp53^R172H^ model of pancreatic cancer, which demonstrated NLRP3 signaling in macrophages to drive M2 polarization and suppress both the activation and infiltration of CD4^+^ and CD8^+^ T cells in pancreatic tumors [43]. This data is further in line with findings implicating macrophage NLRP3 signaling to enhance the migration and metastatic progression in models of both colorectal cancer and melanoma [44,45]. Additional studies have shown NLRP3 expression by MDSCs to mitigate against the anti-tumor properties of 5-fluorouracil chemotherapy in several preclinical tumor models by driving T_H_17 development [46]. Notably, these effects were both found to be IL-1β-dependent. Indeed, many of the described pro-tumorigenic properties of NLRP3 have been attributed to its role in driving the expression of IL-1β by tumor-infiltrating myeloid cells. Studies have shown this process to support the recruitment of other myeloid cells to the tumor bed and promote tumor invasiveness and metastasis in an IL-1β-dependent manner [45,47]. Indeed, studies have implicated the NLRP3-mediated release of IL-1β in the induction of the IL-22 cytokine by CD4^+^ T cells, a process observed to support tumor cell proliferation and growth [48]. Together, these data are consistent with the findings reported in the CANTOS clinical trial, which was originally designed to examine the impact of the IL-1β antagonistic antibody, canakinumab, on recurrent vascular events in patients with a prior myocardial infarction and persistently elevated C-reactive protein. Remarkably, a re-evaluation of this data revealed a significant decrease in lung cancer incidence (HR 0.33, *p* < 0.0001) and lung cancer mortality (HR 0.23, *p* = 0.0002) in patients treated with canakinumab relative to placebo [49].

While several studies have demonstrated IL-1β to generally promote both intrinsic and extrinsic properties of tumorigenesis, including angiogenesis and immune evasion, it is important to also recognize that a role for IL-1β has been described in promoting anti-tumor immunity in specific contexts. This includes a report describing the delivery of systemic IL-1β distant from the tumor site to effectively condition adoptively transferred T cell populations to generate improved anti-tumor immune responses in a B16 melanoma model [50]. Additional groups utilizing syngeneic murine tumor model systems have reported on the anti-tumor properties of IL-1β [51,52]. Indeed, IL-1β signaling in dendritic cells has been shown to be critical for the induction of radiation-induced anti-tumor immune responses [53]. The exact underlying reason for this seemingly discrepant data is generally believed to be associated with the context of IL-1β signaling, while the local concentration and secretion kinetics of IL-1β may also influence downstream outcomes following the activation of this pathway. These disparate results further emphasize the importance of validating pre-clinical data with correlative studies in cancer patients.

## 5. Tumor-Intrinsic NLRP3 and Adaptive Immunotherapy Resistance

**Figure 1:**
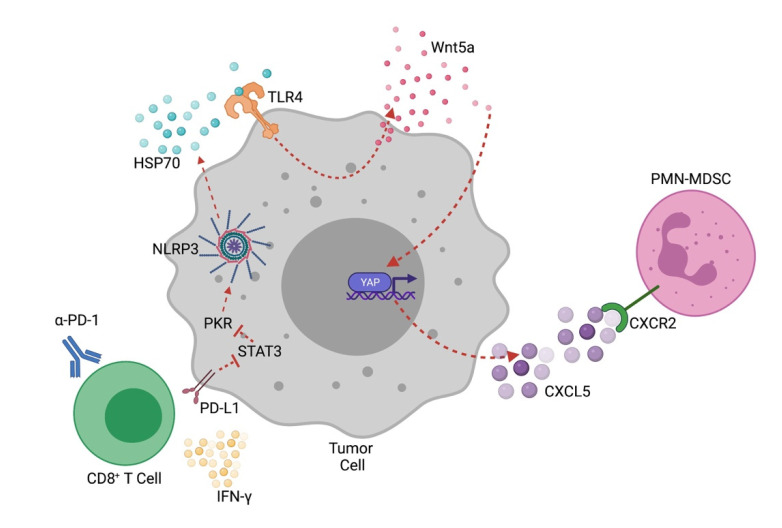
Tumor-Intrinsic NLRP3 Inflammasome Promotes Adaptive Resistance to Anti-PD-1 Immunotherapy. A PD-L1:STAT3:PKR pathway triggers NLRP3 inflammasome activation in response to anti-PD-1-mediated CD8^+^ T cell activation [54]. In line with previous work, our studies have shown that PD-L1 cytoplasmic signaling inhibits STAT3 and that this process de-inhibits PKR kinase activity to lead to NLRP3 activation [60,61]. A TLR4-Wnt5a-CXCL5 autocrine pathway promotes PMN-MDSC recruitment in response to tumor NLRP3-dependent release of HSP70. Created with BioRender.com.

While the above studies are consistent with the previously described pro-tumorigenic properties of the NLRP3 inflammasome, a role for tumor-expressed NLRP3 in the regulation of anti-tumor immunity has remained less clear. Using a transgenic model of BRAF^V600E^ melanoma, we found anti-PD-1 immunotherapy to induce the upregulation of several CXCR2-dependent chemokines in primary melanoma tissues [54] (Figure 1). We verified that these changes correlated with an increased flux of PMN-MDSCs into tumors, a process that was reversed by tumor-directed genetic silencing of CXCL5 as well as small molecule inhibition of CXCR2. This observation was further found to correlate with the upregulation of Wnt5a expression in tumor tissues in response to anti-PD-1 therapy [54]. Building on prior key studies, we found this upregulation in Wnt5a to drive both CXCL5 expression and the recruitment of PMN-MDSCs in response to anti-PD-1 [55,56]. While conducting this work, we also noted that tumors undergoing CD8^+^ T cell-mediated killing in response to anti-PD-1 immunotherapy exhibited an upregulation in several cellular stress pathways, including the release of HSP70. Since HSP70 has been shown to induce toll-like receptor-4 (TLR4) activation, which has further been linked with Wnt5a upregulation, we surmised that the release of HSP70 in response to PD-1-PD-L1 blockade could be inducing this Wnt5a-CXCL5 signaling axis [57,58]. Indeed, additional studies demonstrated that HSP70 was readily secreted from these melanoma cells in response to CD8^+^ T cell activation both *in vitro* as well as *in vivo* [54]. Adenosine triphosphate (ATP) has been demonstrated to be a potent inducer of HSP70 secretion [59]. Since ATP is also a well-known mediator of NLRP3 inflammasome activation in several cell types, we hypothesized that the NLRP3 inflammasome was mediating the release of HSP70 in response to the anti-PD-1 blockade. Indeed, additional experiments both *in vitro* and *in vivo* using both genetic silencing and pharmacologic inhibition demonstrated the release of tumor HSP70 to be dependent on activation of the tumor-intrinsic NLRP3 inflammasome [54]. We further confirmed and characterized this same overall pathway in an array of murine tumor models and human tumor cell lines that included pancreatic cancer, colorectal cancer, non-small cell lung cancer, and renal cell carcinoma. These data suggested that this observed pathway might be related to a unique activation mechanism for the NLRP3 inflammasome in several tumor types. Therefore, a series of additional signaling studies was conducted to further define a link between CD8^+^ T cell activation and NLRP3 inflammasome activation in tumors, revealing tumor PD-L1-mediated triggering of NLRP3 activation via a STAT3-protein kinase R (PKR) pathway (Figure 1) [54,60,61]. This work further showed CD8^+^ T cell-derived interferon-γ to contribute to the activation of this pathway, likely through the upregulation of PD-L1 expression. Together, these data outline a role for the tumor-intrinsic NLRP3 inflammasome in driving acquired resistance to checkpoint inhibitor immunotherapy by triggering the recruitment of an immunosuppressive population of PMN-MDSCs. Additional studies showing combination NLRP3 inhibition-anti-PD-1 antibody therapy to generate a more effective anti-tumor immune response over anti-PD-1 monotherapy in the autochthonous BRAF^V600E^ melanoma model further support the importance of this tumor-intrinsic pathway in mediating resistance to checkpoint inhibitor immunotherapy.

## 6. Monitoring Tumor-Intrinsic NLRP3

This work is consistent with recently reported findings portraying a positive relationship between an NLRP3-associated gene expression signature and tumor-infiltrating lymphocytes based on a review of the melanoma SKCM database in the Cancer Genome Atlas (TCGA) [62]. However, this study also noted an association between elevated NLRP3 gene expression scores and a positive prognosis in melanoma patients [62]. This contradicts the findings of several studies described above, indicating that NLRP3 supports tumorigenesis and immune evasion through both intrinsic and extrinsic mechanisms. We propose that evidence of an immunosuppressive impact by activation of the NLRP3 inflammasome will not be entirely apparent until the introduction of an anti-PD-1 antibody. Indeed, the majority of TCGA samples were derived from primary and regional metastatic lesions, all prior to the initiation of checkpoint inhibitor immunotherapy [63]. In addition, intrinsic negative feedback expression loops in this pathway are likely to complicate interpretation of the NLRP3 gene expression score for specific tissues based on transcriptional profiling, as high NLRP3 gene expression scores are likely to be associated with lower NLRP3 signaling activity [64]. Finally, various post-transcriptional modifications of NLRP3 are expected to alter NLRP3 signaling capacity, further making transcriptional measurements on the RNA level a poor estimate of the activity of this pathway [4]. This further emphasizes the importance of the use of functional assays, such as NLRP3-ASC coaggregation assays in tissues or the measurement of other downstream targets upregulated by NLRP3, rather than relying on transcriptional studies for assessing the activity of the NLRP3 inflammasome.

## 7. Genetics of Tumor-Intrinsic NLRP3

**Figure 2:**
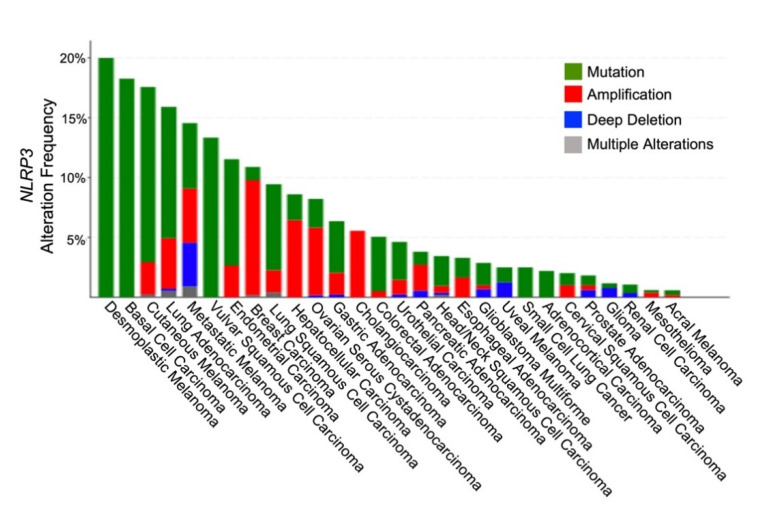
NLRP3 Genetic Alterations in Various Cancer Types. Figure adapted from cBioportal.com.

There has been a plethora of studies associating various gain-of-function mutations in *NLRP3* with several autoinflammatory syndromes, and this has been supported by mechanistic studies illustrating a direct causal relationship between specific *NLRP3* mutations and inflammatory sequelae [1,2,65]. In addition, a review of the TCGA database also shows an array of cancer types harboring genetic alterations in *NLRP3* (Figure 2) [65,66]. Nevertheless, our understanding of the influence of genetic variation in NLRP3 on tumor behavior and tumor immunosurveillance remains poor. Whether germline genetic alterations contribute significantly to tumor-intrinsic NLRP3 functionality or whether the activation of tumor-intrinsic NLRP3 is dominantly controlled by tumor somatic mutations also remains unclear. As noted previously, gene amplification in *NLRP3* has been identified in select cancer types, including breast cancer, ovarian cancer, and melanoma [24]. This is further consistent with the observation that several cancer types exhibit an upregulation in the expression of NLRP3 relative to their benign tissue counterparts [67]. Specific small nucleotide polymorphisms (SNPs) of *NLRP3* have been identified in the genome of patients and associated with the development and/or prognosis of certain malignancies, including melanoma and colorectal cancer [68,69]. However, mechanistic studies explaining the relationships between these SNPs and enhanced tumorigenesis are still lacking. Notably, recent studies in lung adenocarcinoma have revealed select gain-of-function mutations in NLRP3 to drive FLIP-dependent inhibition of apoptosis, thus promoting tumorigenesis [25]. It would be interesting to further determine if these NLRP3 mutant lung tumors were also associated with enhanced recruitment of MDSC populations.

In light of its emerging role in regulating anti-tumor immunity, genetic alterations in NLRP3 may predict targeted pharmacologic inhibitors of NLRP3 to augment checkpoint inhibitor immunotherapy. As a variety of genetic alterations, including gain-of-function mutations and copy number gains, may contribute to the overall activation state of NLRP3 in tumors, functional NLRP3 activation assays, such as NLRP3-ASC co-localization studies, using tumor tissue specimens may serve as a surrogate capable of capturing those tumors utilizing NLRP3 to suppress immune surveillance. Our data showing tumor-intrinsic NLRP3 to drive HSP70 release further suggests the use of plasma HSP70 levels as another candidate predictive biomarker for either NLRP3 or HSP70-targeted inhibitors. Finally, since specific NLRP3-related mutations may dictate the efficacy of select inhibitors, the identification of those mutations impacting NLRP3 function may be critical for the selection of pharmacologic inhibitors to manage specific tumors [70].

## 8. Role of HSP70 as a Mediator of Tumor-Intrinsic NLRP3

**Figure 3:**
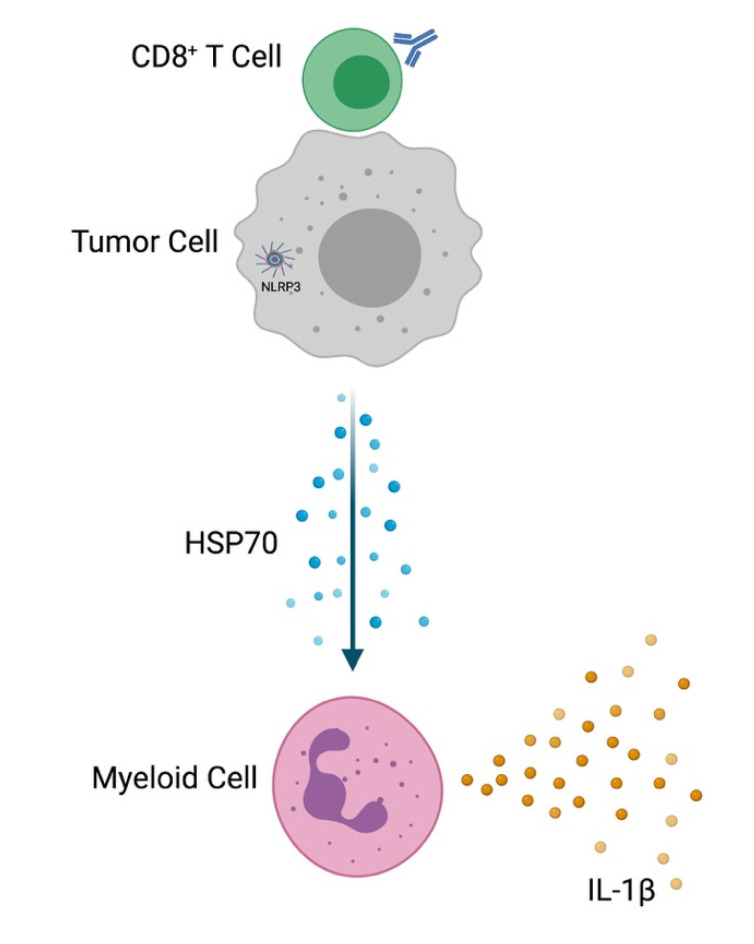
Tumor-intrinsic NLRP3 Inflammasome Mediates Release of HSP70 From Tumor Cells. In turn, accumulated myeloid cells, such as PMN-MDSCs, in the tumor microenvironment produce elevated levels of IL-1β. Created with BioRender.com.

The HSP70 family is comprised of 13 proteins that reside within different cellular compartments and generally serve to support cell survival during various forms of cellular stress [71]. Several mechanisms have been proposed to explain the secretion of HSP70, which has been shown to be independent of the endoplasmic reticulum and the Golgi [71]. In comparison to the levels of IL-1β typically generated via NLRP3 activation in myeloid cell populations, the levels of IL-1β secretion via the tumor-intrinsic NLRP3 pathway in both murine melanoma models and in human melanoma cell lines are minimal. Rather, our studies implicate HSP70 as being the critical soluble mediator released by the tumor NLRP3 inflammasome as well as the driver of PMN-MDSC recruitment in several murine models and human tumor cell lines [54]. Indeed, we further identified patients with anti-PD-1-resistant melanoma to exhibit elevated HSP70 plasma levels as well as an increase in the myeloid-associated genes, *CD33*, *CD11B*, *CXCR2*, *S100A8*, and *S100A9,* in tumor tissue specimens relative to responders [54]. It is notable that this data is consistent with a recent study that also recognized a relationship between the tumor-intrinsic NLRP3 inflammasome and the recruitment of MDSCs in the B16/F10 melanoma model [72]. These authors showed that a selective pharmacologic inhibitor of NLRP3, OLT1177, suppressed B16/F10 melanoma progression and inhibited IL-1β release in the 1205Lu human melanoma cell line. This work also reported an improved anti-tumor immune response when combining OLT1177 with anti-PD-1 antibody therapy in the B16/F10 melanoma model and inferred that this was due to inhibition of melanoma-dependent IL-1β secretion. However, no experiments were presented showing a direct role for IL-1β in the recruitment of MDSCs. Unlike prior studies that have shown NLRP3-dependent release of IL-1β occurs in human melanoma cell lines and tissues derived from more advanced disease, we have been unable to induce IL-1β expression in human melanoma cell lines at significant levels with common NLRP3 stimuli [73]. Indeed, even when detected, the levels of IL-1β produced by melanoma cells are negligible relative to what is observed following the stimulation of various myeloid cell populations. Notably, the use of combination IFN-γ and anti-PD-L1 antibody treatment to simulate CD8^+^ T cell activation-induced HSP70 secretion by these same human melanoma cell lines, while IL-1β remained undetectable. While the preponderance of the data supports an important role for IL-1β in contributing to tumorigenesis, we propose that its role is further downstream from tumor NLRP3-mediated HSP70 release and is predominantly expressed by recruited myeloid cells in the tumor microenvironment as opposed to tumor cells (Figure 3). This described secretion mechanism of HSP70 from tumor cells in response to CD8^+^ T cell cytolytic activity contributes to our understanding of extracellular HSP70. In addition, these findings implicate HSP70 as being an additional candidate immunotherapeutic target with the capacity for synergizing with anti-PD-1 immunotherapy.

## 9. NLRP3 and HSP70 Inhibitors in Development

There are a variety of pharmacologic inhibitors of the NLRP3 inflammasome in early-phase clinical trial testing for several autoimmune and autoinflammatory conditions [74,75]. However, few have been investigated in cancer, and many of these inhibitors have only been tested in pre-clinical studies (Table 1). This includes MCC950, a diarylsulfonylurea-containing compound that inhibits ATP hydrolysis in the NACHT domain of NLRP3, which has been found to suppress MDSCs and regulatory T cells while enhancing the numbers of effector T cells in murine models of head and neck squamous cell carcinoma [42,76]. Ordinine is a covalent small molecule inhibitor derived from the Chinese medicinal herb *Rabdosia rubescens,* which inhibits the assembly of the NLRP3 complex by interacting with the Cys279 amino acid within its NACHT domain [77]. Studies have demonstrated this inhibitor to suppress colon cancer cell proliferation *in vitro* and tumor progression in syngeneic tumor models *in vivo* [78]. Better known for its anti-allergy properties, tranilast (N-3,4-dimethoxycinnamoyl-anthranilic acid), which also binds to the NACHT domain to prevent NLRP3 oligomerization, has been shown to suppress the progression of a variety of cancer types in pre-clinical studies, including gastric cancer, pancreatic cancer, glioma, and osteosarcoma [79]. Notably, dapansutrile (OLT1177), a small synthetic orally active compound and selective NLRP3 inhibitor, which is currently the most advanced candidate in clinical trial development, has been evaluated for the treatment of heart failure, osteoarthritis, as well as acute gout in a recent phase II study [80,81]. In addition to studies focused on the use of dapansutrile for the management of inflammatory diseases, a recent study has shown this agent to suppress the recruitment of MDSCs and enhance anti-tumor immunity in the B16 melanoma model, a finding in line with our previously described data [54,72].

Given that the NLRP3 inflammasome has been implicated in an array of inflammatory conditions, several additional direct NLRP3 inhibitors, some with restricted tissue distribution profiles, are currently in various phases of development [74]. In addition, interest has also been shown in developing indirect NLRP3 inhibitors that primarily target other regulators of the NLRP3 inflammasome. These include NEK7, which is necessary for the induction of NLRP3 oligomerization, JNK1-mediated phosphorylation, as well as several players that regulate NLRP3 levels via ubiquitination, including BRCC3, FBXL2, FBXO3, MARCH7, and TRIM31 [4,7]. Together, these trends suggest the future availability of several candidate inhibitors that may be re-purposed for tumors harboring specific genetic alterations impacting the activity of this pathway. We direct the reader to a comprehensive review of several additional topics related to NLRP3 inhibitor development and application that are beyond the scope of this current review [74].

As studies have continued to implicate HSP70 as playing a role in the process of tumorigenesis, HSP70-targeted inhibitors have also emerged in cancer studies [71]. In particular, VER-155008, which binds to the nucleotide binding site of HSP70, has been shown to suppress the proliferation and promote the apoptosis of several different tumor cell lines, including prostate cancer and lung cancer [82,83]. This is consistent with the observed activity of apoptozole, which also inhibits the ATPase activity of HSP70 and induces the apoptosis of several different cancer cell lines *in vitro* [84]. This is also true for the activity of the synthetic HSP70 inhibitor, MAL3-101, previously demonstrated to suppress proliferation and growth of Merkel cell carcinoma tumor cells *in vitro* and *in vivo*, respectively [85]. While there have been few studies linking the inhibition of HSP70 with anti-tumor immunity, a recent study performed in the MC38 and CT26 murine colon cancer cell lines as well as the HT-29 human colon cancer cell line demonstrated the HSP70 inhibitor, AP-4-139B, to induce an immunogenic form of cell death that promotes the recruitment of both CD4^+^ and CD8^+^ T cells [86]. Finally, another HSP70 inhibitor, minnelide, has also been found to have anti-tumor properties and is currently being investigated in a series of clinical trials focused on various cancer types (Table 1) [87,88].

As opposed to a role as an inhibitor of tumor immunosurveillance, there is existing data describing a supportive role of the NLRP3 inflammasome in the development of anti-tumor immune responses, including data illustrating its ability to promote dendritic cell-dependent activation of T cells [89]. This would be consistent with studies indicating that the NLRP3 inflammasome also plays an important role in the development of immune responses directed toward pathogens, including influenza A [90]. Indeed, prior clinical investigation of the IL-1β antagonistic antibody, canakinumab, did reveal an increase in infectious complications associated with this agent [91]. Furthermore, previous work has identified a T cell-intrinsic role for NLRP3 in directing T_H_2 differentiation in CD4^+^ T cells [92]. These data, therefore, indicate that systemic NLRP3 inhibition should be approached with caution. These data further suggest that HSP70 may represent a more tumor-specific target since our data indicate NLRP3-mediated HSP70 release occurs predominantly in malignant tissues rather than in myeloid cell populations.

## 10. Conclusions

While implicated in driving certain intrinsic tumorigenic properties of cancers, an important role for the NLRP3 inflammasome in directing anti-tumor immunity is now being realized. The contribution of the NLRP3 inflammasome in the context of myeloid cell populations to the development of various pro-inflammatory states has been well documented. However, few studies have sought to characterize the function and regulation of the NLRP3 inflammasome in tumor cells. Studies to date suggest that the tumor-intrinsic NLRP3 inflammasome plays an important role in recruiting the highly immunosuppressive PMN-MDSCs into the tumor microenvironment and facilitating tumor immune evasion as well as resistance to checkpoint inhibitor immunotherapy. An improved understanding of those molecular mechanisms directing NLRP3 activity in tumors as well as the genetic variations that dictate the overall activity of this pathway in various cancers are predicted to lead to novel pharmacologic targets and biomarkers capable of guiding the use of these agents. The future development of tumor-specific NLRP3 pathway antagonists may ultimately prove to be the most effective strategy for enhancing the efficacy of checkpoint inhibitor immunotherapies in clinical oncology.

## Figures and Tables

**Table 1:** Clinical Trial Testing of NLRP3 and HSP70 Inhibitors in Oncology.

Target	Compound Name	Trial Phase	Indication	Status	NCT Identifier
**NLRP3**	Dapansutrile (OLT1177)	I/II	w/ Pembrolizumb in Anti-PD-1-Resistant Melanoma	Not Yet Recruiting	NCT04971499
	Resveratrol	I	Colon cancer	Completed	NCT00256334
	Resveratrol	I	Colon cancer	Completed	NCT00433576
	Resveratrol	I	Colon Cancer	Completed	NCT00920803
**HSP70**	Minnelide	I	Advanced GI Tumors	Completed	NCT01927965
	Minnelide	II	Refractory Pancreatic Adenocarcinoma	Completed	NCT03117920
	Minnelide	II	Refractory Pancreatic Adenosquamous carcinoma	Recruiting	NCT04896073
	Minnelide	I	Relapsed or Refractory Acute Myeloid Leukemia	Recruiting	NCT03760523
	Minnelide	I	Advanced Solid Tumors	Recruiting	NCT03129139

## Data Availability

Not applicable.

## Abbreviations

CAF	cancer-associated fibroblast
CANTOS	Canakinumab Anti-Inflammatory Thrombosis Outcome Study
DAMPs	danger-associated molecular patterns
EMT	epithelial-mesenchymal transition
HSP70	heat shock protein-70
IL-1β	interleukin-1 beta
IL-18	interleukin-18
NACHT	nucleotide-binding and oligomerization
NEK7	never in mitosis A (NIMA)-related kinase 7
NLRP3	NOD-like receptor family, pyrin-domain-containing-3
PD-L1	programmed death-ligand-1
PKR	RNA-dependent protein kinase
PMN-MDSC	granulocytic myeloid-derived suppressor cell
ROS	reactive oxygen species
TCGA	The cancer genome atlas
TLR4	toll-like receptor-4
TME	tumor microenvironment
YAP	Yes-associated protein
